# Educational attainment and deaths of despair among individuals assessed for substance use severity: Findings from Swedish Addiction Severity Index (ASI) data

**DOI:** 10.1177/14550725251326757

**Published:** 2025-03-13

**Authors:** Simone Scarpa, Wossenseged Birhane Jemberie, Björn Högberg, Lena Lundgren

**Affiliations:** Department of Social Work, 8075 Umeå University, Umeå, Sweden; Department of Social Work, 8075 Umeå University, Umeå, Sweden;; Centre for Demography and Aging Research (CEDAR), 8075 Umeå University, Umeå, Sweden; Department of Social Work, 8075 Umeå University, Umeå, Sweden;; Centre for Demography and Aging Research (CEDAR), 8075 Umeå University, Umeå, Sweden; Department of Social Work, 8075 Umeå University, Umeå, Sweden;; Cross-National Behavioral Health Laboratory, 2927 University of Denver, Denver, CO, USA; Graduate School of Social Work, 2927 University of Denver, Denver, CO, USA

**Keywords:** Addiction Severity Index (ASI), deaths of despair, drug use-related mortality, Sweden, education

## Abstract

**Aims:** This study examined the association between educational attainment and the risk of deaths of despair among individuals assessed for substance use severity at municipal social services in Sweden. It aimed to determine whether the protective association between education and despair-related mortality risk, commonly observed in broader population studies, also applies to this high-risk, treatment-seeking population. **Methods:** We linked data from municipal Addiction Severity Index (ASI) assessments to various population registers. The study population included adults aged 25 years or older who were assessed for substance use severity between 1999 and 2019. Fine–Gray competing risk regression models were employed to examine the association between educational attainment and despair-related mortality, both overall and by subtype (alcohol-related, drug use-related, suicide). **Results:** Tertiary education was unexpectedly associated with increased risks of overall despair-related mortality, alcohol-related and drug use-related mortality, after controlling for demographic characteristics, ASI composite scores and substance use onset age. No significant associations were found between education and suicide. **Conclusions:** The counterintuitive finding that tertiary education is linked to a greater risk of despair-related mortality among social service users may be attributed to differences between the treatment-seeking population and the general population. Highly educated individuals who seek treatment from municipal social services might have severe substance use and complex psychosocial problems and/or contact social services after exhausting other treatment options. Further research is necessary to understand how educational and socioeconomic factors influence treatment pathways for individuals with severe substance use problems in Sweden and how these different pathways impact health-related outcomes.

## Introduction

Sweden, comprising a country recognized for its extensive social welfare system and high standard of living, faces a growing challenge related to drug use-related mortality. This issue has intensified over the years, setting Sweden apart from many European counterparts. A recent study revealed that, between 1990 and 2019, Sweden showed a higher increase in Disability-Adjusted Life Years (DALYs) due to drug use-related compared to both European and global averages (Agardh et al., 2023). According to the same study, in 2019, the DALY rate for drug use disorders among males in Sweden was the second highest in the Nordic region, only surpassed by Finland, while the rate among females was below the European average. Cross-country comparisons of drug use-related mortality between Sweden and other European nations are, however, complicated by issues of data comparability. Reports from the European Monitoring Centre for Drugs and Drug Addiction (EMCDDA) have sometimes listed Sweden as having among the highest drug use-related death rates per capita among EU countries (EMCDDA, 2020). Nevertheless, variations in national reporting practices are known to influence the comparability of these data (Waal & Gossop, 2013). For example, Sweden conducts more comprehensive forensic investigations, including toxicological analyses, for unnatural deaths than other countries (Fugelstad, 2022). Despite these data comparability issues, there is broad scholarly consensus on the urgent need to address the severe situation of drug use-related mortality in Sweden, especially given the recent increase in fatalities (Fugelstad, 2022; Goldberg, 2021; Holmén et al., 2023). A recent collaborative report by several Swedish governmental agencies not only acknowledged the discrepancies in national data used by international organizations such as the World Health Organization and the EMCDDA, but also emphasized that these discrepancies should not overshadow the alarming levels of drug use-related mortality in the country (Folkhälsomyndigheten et al., 2022).

Of particular concern in Sweden is the disproportionate impact of drug use-related mortality on young adults, especially due to overdoses of opioids, including fentanyl and buprenorphine (Folkhälsomyndigheten, 2024; Folkhälsomyndigheten et al., 2022; Fugelstad, 2022). Sweden records the highest number of such deaths in the Nordics (Simonsen et al., 2020). A recent study (Ågren & Bremberg, 2022) found that mortality related to drug use, particularly opioid use, along with other forms of self-harm such as suicides, was a leading contributor to mortality among Swedish young adults (aged 20–34 years) between 2000 and 2017. According to Ågren & Bremberg (2022), these issues contributed to the stagnation of the mortality decline within this demographic, which is a trend that diverges from patterns observed among other Swedish age groups and among similarly aged European peers. Ågren and Bremberg (2022) further suggested that the possible cause behind this trend may be the deteriorating living conditions for young adults in Sweden, particularly due to significant penalties for those not completing upper-secondary education, which in turn hinders continuation to tertiary education and negatively impacts labour market outcomes. Subsequent research by Högberg et al. (2025) has substantiated these findings, documenting a rise in mortality from preventable causes, including drug use and suicide, among low academically achieving youth from 1990 to 2018.

In Swedish policy and academic debates, this explanation has drawn parallels (Ågren & Bremberg, 2023; Högberg et al., 2025) with “deaths of despair” in the USA, a term originally coined to describe rising mortality rates among middle-aged white Americans without college degrees, encompassing deaths from drug overdoses, alcohol-related diseases and suicide (Case & Deaton, 2015, 2020). In Sweden, the affected demographic primarily includes young adults with lower educational attainment, suggesting a different set of socioeconomic dynamics (Ågren & Bremberg, 2022; Folkhälsomyndigheten et al., 2022). Education, within the “deaths of despair” framework, is viewed as a protective factor that can buffer individuals against the sense of hopelessness associated with labour market disadvantage, which may lead to risky substance use and other related or unrelated self-harming behaviours (Case & Deaton, 2020). However, the interplay between educational attainment and deaths of despair is complex and appears to vary across different national contexts (Lübker & Murtin, 2024).

The present study aims to investigate the relationship between educational attainment and despair-related mortality within a high-risk population in Sweden: individuals who have been assessed for substance use severity at municipal social services. Focusing on this clinical sample allows us to provide targeted insights into a group with complex social and healthcare needs. Identifying risk and protective factors specific to these social service clients is crucial for informing interventions aimed at reducing mortality. We aim to determine whether the protective association between educational attainment and mortality risk, commonly observed in broader population studies, also applies to social service users with substance use problems. We extend previous research on mortality risks within this treatment-seeking population (Lundgren et al., 2019; Lundgren et al., 2022; Scarpa et al., 2023) by examining alcohol-related, drug use-related and suicide-related mortality as competing risks. Employing Fine–Gray subdistribution hazard models (Fine & Gray, 1999), we analyze the association between educational attainment and each of these competing risks.

### The protective role of education against deaths of despair

Introduced by economists Case and Deaton (2015, 2020), the term “deaths of despair” encompasses mortality caused by drug overdoses, suicides and alcohol-related conditions. These scholars used the term to explain the significant rise in mortality rates among middle-aged (45–54 years), non-Hispanic white Americans during the 2000s, marking a reversal of a century-long decline. At the heart of this shift is the role of education, or rather the lack thereof. Indeed, this alarming trend has predominantly impacted individuals without a college degree. Diminished job prospects for these individuals are hypothesized to lead to worsening living conditions and a pervasive sense of hopelessness, fostering self-harming behaviours, including risky substance use, suicidality and increased mortality risks from these specific causes.

The association between low educational attainment and detrimental health outcomes, including reduced life expectancy, is well established in the literature (Consolazio et al., 2024; Raghupathi & Raghupathi, 2020; Schnittker, 2004). This stream of research also encompasses specific findings on substance use-related health problems (Armoon et al., 2023; Fishman & Gutin, 2021; Link & Phelan, 1995). Adding to this field, the “deaths of despair” literature highlights the widening educational gradient in mortality in recent decades, specifically pinpointing increases in suicides, drug use-related and alcohol-related fatalities. This widening gradient, where individuals with lower educational attainment face increasingly severe disadvantages, results from shifts in labour market dynamics that demand high human capital competencies (Bjorklund, 2023; Case & Deaton, 2020). Such exacerbation of the educational gradient can affect demographic developments related to the life expectancy of various groups across national contexts (King et al., 2022; Lübker & Murtin, 2024).

Recent studies have examined how educational attainment serves as a protective barrier against deaths of despair. Due to limitations in data availability, individual-level analyses are rare. Park (2024), for example, analyzed data from a Canadian nationally representative dataset for the period 2011–2018 and found that low educational attainment, particularly the absence of post-secondary education, combined with other socioeconomic factors such as low income or unemployment, was significantly associated with a higher risk of all types of deaths of despair. Using Spanish data from mortality registers from 1980 to 2019, Piñeiro et al. (2023) observed an overall decrease in all causes of despair-related mortality, a trend contrasting with those observed in the USA and other countries. Piñeiro et al. (2023) specifically examined educational disparities in despair-related mortality from 2017 to 2019 and found that these disparities are more pronounced among men than women, consistent with patterns observed in the USA (Case & Deaton, 2020).

The easier accessibility to geographically aggregated data explains why most studies have an ecological perspective. For example, Siddiqi et al. (2019), using US county-level data, found that the rise in despair-related mortality rates between 2000 and 2016 affected a wider demographic than initially identified by Case & Deaton (2020), encompassing individuals aged 25–54 years, with low educational attainment being a common risk factor across age groups. Schnake-Mahl et al. (2024) focused on the state of Pennsylvania and found that the rate of mortality for despair-related causes was higher in congressional districts where a larger share of the population had attained no more than a high school education. Furthermore, Dow et al. (2020) used geocoded data (for the period 1999–2017) in a quasi-experimental design during a labour market policy reform introduced across some US states between 2007 and 2009. They compared the risk of deaths of despair before and after these state-level policy changes and found that improvements in income reduced suicides but did not affect drug use-related fatalities among individuals without tertiary education.

Adding a cross-country comparative perspective, Lübker and Murtin (2024) analyzed data for individuals aged over 25 years from 14 selected OECD (i.e., Organisation for Economic Co-operation and Development) countries in the period 2013–2019 to estimate the impact of deaths of despair on general life expectancy and the life expectancy gap between educational groups. They found that deaths of despair had a comparatively stronger impact on both general life expectancy and life expectancy gaps between educational groups in certain countries (not only the USA, but also Sweden), and a minimal impact in other countries, where it was also more uniformly distributed across educational attainment groups (e.g., Southern European countries).

To our knowledge, the study by Dahlman et al. (2021) is the only one based on Swedish data that focused on the protective role of education against drug use-related mortality, namely opioid overdoses, considering both non-fatal incidents and fatal outcomes. Using register data from 2015 and 2017, they found that education, measured in years, had a protective effect against incident overdoses in both bivariate and multivariate analyses. However, this protective effect against fatal outcomes was statistically significant only in the bivariate analyses.

By contrast, the link between education and suicide risk is more established and frequently examined in Swedish research. For example, Döring et al. (2021) reported that failing to qualify in upper-secondary school was associated with an increased risk of suicide and other causes of premature mortality up to middle age. Similarly, Sörberg Wallin et al. (2020) noted that educational attainment partially explained the relationship between academic performance and suicide attempts, and Gunnell et al. (2011) found a strong inverse relationship between school performance and suicide risk among males. However, the findings from these studies are based on older cohorts from the 1970s, and it remains unclear how applicable they are to contemporary populations.

We make two key contributions to previous Swedish research on education and despair-related mortality. First, we analyze all subtypes (i.e, suicide, drug- and alcohol-related mortality) together, thereby providing a more comprehensive picture of the association between education and despair-related mortality. Second, we use data on individuals assessed for substance use severity at municipal social services, thereby allowing us to test whether findings from the general population are also applicable to a high-risk, treatment-seeking population. In doing so, our study not only enhances understanding of these relationships, but also offers insights that may inform the development of more targeted interventions for this vulnerable group.

## Methods

### Study design

This retrospective cohort study examined the association of educational attainment with despair-related mortality, both as a collective category and through its specific components. The study involved individuals assessed for substance use severity at Swedish municipal social welfare offices from 1999 to 2019. Participants were followed until death or the conclusion of the study period in December 2019, which was treated as right-censoring for the analysis.

### Study setting and data sources

In Swedish municipal social services, social workers trained on the Addiction Severity Index (ASI) (McGahan et al., 1986; McLellan et al., 1992) use this tool to assess clients’ substance use severity and evaluate intervention needs across various biopsychosocial life domains. These domains include physical health, employment, drug use, alcohol, legal problems, family/social relations and mental health. Based on a standardized questionnaire, each domain's composite score is calculated by weighting and summing client responses on a scale from 0 (minimal issues) to 1 (severe problems) (McGahan et al., 1986; McLellan et al., 1992). According to national guidelines, the ASI assessment is specifically designed for clients with suspected or confirmed substance use problems and is not routinely administered to individuals without such issues. The assessment is typically conducted during the second meeting with the client (after being introduced to the client in the first meeting). However, in acute cases, the assessment is deferred until the client's situation has stabilized after initial intervention.

Swedish municipalities have increasingly adopted the ASI as the primary assessment tool for substance use and related problems, with adoption rates rising from less than one-third of municipalities to over 90% (Jemberie et al., 2022; Lindner et al., 2024; Scarpa et al., 2024; Tengvald et al., 2004). This study employed baseline ASI assessment data from 89 Swedish municipalities, which were linked at the individual level to the Swedish Cause of Death Register from the Swedish National Board of Health and Welfare (NBHW) (Brooke et al., 2017) and the Total Population Register from Statistics Sweden (SCB) (Ludvigsson et al., 2016). The study population thus consists of individuals with varying degrees of substance use severity, as assessed by a standardized and validated tool.

### Study population

**Figure 1.**
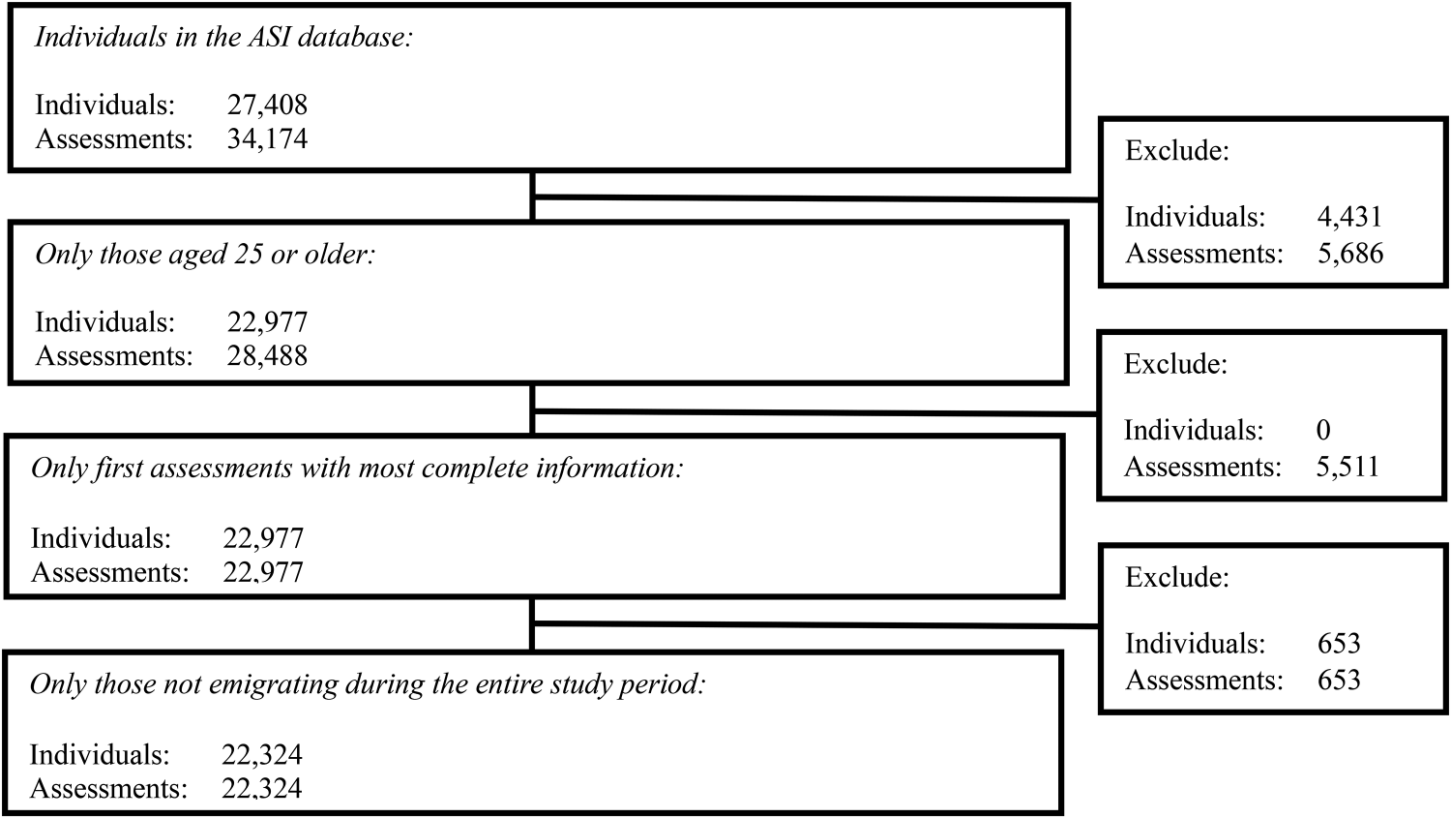
CONSORT flow diagram for the study population. ASI =  Addiction Severity Index.

Our study covered the years 1999 to 2019 and focused on individuals aged 25 years or older at the time of their initial assessment, who were residing in Sweden and assessed for the severity of their substance use at municipal social welfare offices using the ASI. We excluded individuals below the age of 25 years, following the approach by Case and Deaton (2017). In Sweden, adults aged 25 years or older are generally considered to be established in the labour market and are no longer eligible for youth-specific labour market programmes (OECD, 2016). For the almost totality of cases (99.5%), the first ASI assessment was used. For participants with multiple ASI assessments, we considered the most complete assessment within 6 months from the initial assessment when the first ASI assessment contained a significant number of missing values on key variables or covariates. This 6-month cutoff aligns with ASI guidelines, which recommend conducting follow-up assessment interviews 6 months after the initial assessment, using slightly different questions from the baseline interview (Socialstyrelsen, 2017). Individuals who emigrated from Sweden after their initial ASI assessment were excluded from the study due to incomplete mortality data. The ASI data spanned from 22 October 1999 to 31 December 2019. Participants were followed until death or the end of the study in December 2019. The Cause of Death Register provided information on underlying and contributing causes of death from 7 March 2000 to 16 September 2019, while the Total Population Register supplied dates of emigration for those excluded from the study. Figure 1 details the inclusion and exclusion criteria used to select the final sample of 22,324 individuals from the initial cohort. For the overall sample, the mean follow-up duration was 2275.5 days (SD = 1536.1 days; median = 2113 days). Among participants who died during the study period, the mean follow-up duration was 1757.8 days (SD = 1277.1 days; median = 1515.5 days).

### Dependent variable

The main outcome measures investigated in this analysis included binary measures of alcohol-related mortality, drug use-related mortality and suicide. Each measure was operationalized based on International Classification of Diseases, 10th Revision (ICD-10) codes from the Cause of Death register, consistent with prior research on deaths of despair (Bjorklund, 2023; Case & Deaton, 2020; Park, 2024), as outlined in Table 1. The three outcome measures were further grouped as an aggregated deaths of despair measure for a separate analysis, while non-despair-related causes of mortality were grouped into another separate category.

**Table 1.** Classification of deaths of despair by International Classification of Diseases, 10th Revision (ICD-10) codes.

Specific category	Subcategory	ICD-10 codes
Alcohol-related mortality	Intentional, unintentional, or undetermined poisoning due to alcohol	X45, Y15
	Mental/behavioural disorders due to alcohol	F100 to F109
	Long-term alcohol-induced illnesses	E244, G312, G621, G721, I426, K292, K700 to K704, K709, K852, K860, R780, Z721
Drug use-related mortality	Intentional, unintentional, or undetermined poisoning due to drugs	X40 to X44; Y10 to Y14; Y45, Y47, Y49; R781 to R785; T400 to T409; T410 to T415; T420 to T428
	Mental/behavioural disorders due to drug	F110 to F119; F120 to F129; F130 to F139; F140 to F149; F150 to F159; F160 to F169; F180 to F189; F190 to F199
	Long-term drug-induced illnesses	D521, D590, D592, D611, D642, E064, E160, E231, E242, E273, E661, G211, G240, G251, G254, G256, G444, G620, G720, I952, I427, J702, J703, J704, K853, L105, L270, L271, M102, M320, M804, M814, M835, M871, R502.
Suicide		X60 to X84; Y870

Both underlying and contributing causes of death were included in the operationalization of the outcome measures. Initially, we checked whether the underlying cause of death matched any of the ICD-10 codes associated with deaths of despair according to our classification. This condition was met for 1247 individuals, accounting for 76.6% of despair-related fatalities in our dataset. Where the underlying cause did not match, we examined the contributing causes, identifying 303 individuals (18.6% of despair-related fatalities) whose underlying cause of death was not linked to despair, but who had a contributing cause that was. Additionally, for 79 individuals (4.8%), multiple contributing causes were identified that linked to various types of despair-related deaths. In these instances, we employed an arbitrary ranking criterion based on the first listed contributing cause in our database.

### Independent variables

The independent variable in this study is educational attainment, operationalized in two ways based on information from the ASI assessments. While the ASI primarily assesses the biopsychosocial impacts of substance use and does not explicitly focus on educational attainment, we have chosen to focus on this variable due to its documented association with health outcomes. This decision aligns with previously discussed research linking socioeconomic factors to despair-related mortality risks, and specifically our study's focus on the relationship between education and mortality risk among individuals assessed for substance use severity.

Educational attainment is operationalized as a categorical variable, self-reported *Educational Level*, which classifies the highest level of education attained into three categories: (1) up to compulsory education (typically up to 9 years, International Standard Classification of Education (ISCED) level 2); (2) upper-secondary education (usually 3 additional years, ISCED levels 3–4); and (3) tertiary education (ranging from 3–5 or more years, ISCED levels 5–8). This categorical operationalization allows for the possibility of non-linear relationships between educational attainment and mortality outcomes, where specific educational levels may be associated with differential risks. The variable is treated as time-invariant, captured at the time of the ASI assessment and reported by the client at the assessment. Additionally, the ASI assessment contains information on the total number of years of education, which we used to operationalize a continuous variable, *Years of Education*. To complement the analysis with the categorical variable, we conducted a sensitivity analysis to test the existence of a linear relationship between years of education and mortality, where each additional year of education may be proportionally associated with mortality risk.

### Covariates

In the present study, we included covariates that are associated with both education and death of despair. Demographic characteristics from the Total Population Register included *Age*, categorized into groups of 25–34 years, 35–49 years and 50+ years; *Sex*, identified as male or female; and *Immigrant Background*, classified into three groups: natives (individuals born in Sweden to Swedish parents), first-generation immigrants (those born outside Sweden) and second-generation immigrants (born in Sweden with at least one parent born abroad). We assessed the severity of client issues across key biopsychosocial domains using seven domain-specific *ASI Composite Scores*, which range from 0 (minimal issues) to 1 (severe problems). These domains included mental health, family/social relations, employment, alcohol use, drug use, physical health and legal issues. The ASI scores are derived from items that focus on clients’ immediate living circumstances, providing a snapshot of their challenges at the time of assessment. Additional alcohol and drug use-related variables included *Onset Age for Heavy Drinking* and *Onset Age for Regular Drug Use*, which were derived from clients’ response during ASI assessment. These variables were included based on their hypothesized association with long-term health outcomes and educational attainment. Early onset of substance use is a known risk factor for prolonged engagement in risky behaviours, which can contribute to health problems and increase mortality risk (Clark et al., 2008; Hu et al., 2017). Furthermore, early substance use initiation may disrupt educational trajectories, leading to lower educational attainment (Grant et al., 2012; Silins et al., 2015). The onset variables were categorized as under 15 years, 15–17 years, 18–24 years and over 24 years. Additional category “no alcohol/drug use” was added for clients who did not report heavy drinking or regular drug use during assessment. In cases where there was a self-reported history of multiple drug use, the onset age for regular drug use was based on the earliest reported onset age during the ASI assessment.

### Statistical analysis

We first compared the characteristics of individuals who died after their ASI assessment with those who survived, using t-tests or chi-square tests to examine potential differences. We used a competing risk regression framework in order to investigate the association of educational attainment with despair-related mortality. This approach was deemed appropriate since deaths from one cause (e.g., alcohol) preclude others (e.g., overdose and suicide). Our study extends previous research (Lundgren et al., 2019; Lundgren et al., 2022; Scarpa et al., 2023) by examining educational attainment's association with various types of despair-related mortality (i.e., categorized as alcohol-related, drug use-related, suicide-related and other non-despair-related causes) using separate Fine–Gray subdistribution hazard models. This approach accounts for the interdependencies among these competing events (Fine & Gray, 1999). We conducted sensitivity analyses using alternative model specifications based on the numeric variable for clients’ *Years of Education* at the time of assessment. Additionally, we performed sensitivity analyses stratified by age group to assess the potential moderating role of age in mortality risk, given that individuals with higher education tend to be older.

## Results

### Sample characteristics

 Table 2 provides the descriptive statistics for the study sample. By the end of the study period on 31 December 2019, approximately 13% of the cohort (*N* = 2902) had died. Among the despair-related causes of mortality, drug use-related deaths were the most prevalent (*N* = 732), followed by alcohol-related deaths (*N* = 675) and suicides (*N* = 221).

**Table 2.** Baseline characteristics and survival status of the study population at end of study period.

Variable	Still living (*n* = 19,422), % or mean (SD)	Deceased (*n* = 2902), % or mean (SD)	Total (*n* = 22,324), % or mean (SD)	*p*-value	Missing (%)
Age at ASI assessment	41.5 (12.0)	49.1 (12.3)	42.4 (12.3)	<0.001	0/22,324 (0.0)
Age group (years)				<0.001	0/22,324 (0.0)
25–34	6,994 (36.0)	472 (16.3)	7,466 (33.4)		
35–49	7,162 (36.9)	911 (31.4)	8,073 (36.2)		
50 or older	5,266 (27.1)	1,519 (52.3)	6,785 (30.4)		
Sex				<0.001	0/22,324 (0.0)
Male	13,600 (70.0)	2,255 (77.7)	15,855 (71.0)		
Female	5,822 (30.0)	647 (22.3)	6,469 (29.0)		
Immigrant background					
Native	13,148 (69.4)	2,149 (75.5)	15,297 (70.2)		
1st generation	2,825 (14.9)	383 (13.5)	3,208 (14.7)		
2nd generation	2,974 (15.7)	314 (11.0)	3,288 (15.1)		
Years of education	11.5 (2.7)	11.0 (2.8)	11.4 (2.7)	<0.001	755/22,324 (3.4)
Education level				<0.001	3,326/22,324 (14.9)
Compulsory or below	6,649 (39.4)	911 (43.3)	7,560 (39.8)		
Upper-secondary	8,055 (47.7)	882 (41.9)	8,937 (47.0)		
Tertiary	2,189 (13.0)	312 (14.8)	2,501 (13.2)		
Onset age of regular drug use (years)				<0.001	1,886/22,324 (8.4)
No regular drug use	5,309 (29.8)	958 (36.6)	6,267 (30.7)		
Below 15	2,567 (14.4)	389 (14.9)	2,956 (14.5)		
15–17	3,033 (17.0)	374 (14.3)	3,407 (16.7)		
18–24	3,752 (21.1)	400 (15.3)	4,152 (20.3)		
Above 24	3,159 (17.7)	497 (19.0)	3,656 (17.9)		
Onset age of heavy drinking (years)					
No heavy drinking	4,173 (23.5)	436 (16.6)	4173 (23.5)	<0.001	1,926/22,324 (8.6)
Below 15	1,369 (7.7)	196 (7.5)	1369 (7.7)		
15–17	2,384 (13.4)	350 (13.3)	2384 (13.4)		
18–24	3,970 (22.3)	470 (17.9)	3970 (22.3)		
Above 24	5,876 (33.1)	1,174 (44.7)	5876 (33.1)		
ASI composite scores					
Mental health	0.336 (0.239)	0.281 (0.237)	0.329 (0.239)	0.000	1,990/22,324 (8.9)
Family/social relations	0.276 (0.229)	0.225 (0.207)	0.270 (0.227)	0.000	1,689/22,324 (7.6)
Legal problems	0.122 (0.212)	0.098 (0.198)	0.119 (0.211)	0.000	1,608/22,324 (7.2)
Drug	0.125 (0.154)	0.094 (0.141)	0.121 (0.153)	0.000	3,234/22,324 (14.5)
Alcohol	0.333 (0.303)	0.375 (0.302)	0.338 (0.303)	0.000	2,318/22,324 (10.4)
Employment	0.754 (0.300)	0.801 (0.261)	0.760 (0.295)	0.000	1,599/22,324 (7.2)
Physical health	0.371 (0.344)	0.430 (0.349)	0.379 (0.345)	0.000	807/22,324 (3.6)
Mortality types					19,422/22,324 (87.0)
Alcohol-related		675 (23.3)			
Drug use-related		732 (25.2)			
Suicide		221 (7.6)			
Other		1,274 (43.9)			

ASI =  Addiction Severity Index.

Compared to those who survived, the individuals who died during the study period were generally older, included a higher proportion of men and had a lower representation of immigrants. Additionally, there was a greater proportion of individuals at both the lowest and highest levels of education among the deceased compared to the surviving group. On average, the deceased had fewer years of education.

### Survival outcomes

 Table 3 presents the adjusted models estimating subdistribution hazard ratios (SHRs) for the general despair-related mortality, alcohol-, drug- and suicide-related deaths. In these models, the independent variable was educational level. Crude SHRs for all included covariates are shown in supplemental Table A1.

**Table 3.** Adjusted Fine–Gray subdistribution hazard models: despair-related mortality, alcohol-related mortality, drug use-related mortality, suicide mortality and non-despair-related mortality as competing risks (adjusted subdistribution hazard ratio with 95% confidence intervals).

Variables	Total despair-related mortality	Despair-related mortality subtypes			Non-despair-related mortality
		Alcohol-related mortality	Drug use-related mortality	Suicide mortality	
Education level					
Compulsory or below	1 (Ref.)	1 (Ref.)	1 (Ref.)	1 (Ref.)	1 (Ref.)
Upper-secondary	1.16 (1.00–1.36)	1.13 (0.87–1.46)	1.18 (0.95–1.47)	1.18 (0.79–1.76)	0.87 (0.73–1.04)
Tertiary	1.45 (1.16–1.82)**	1.46 (1.06–2.01)*	1.63 (1.13–2.34)**	1.08 (0.57–2.06)	0.82 (0.63–1.05)
Age group (years)					
50 yrs or older	1 (Ref.)	1 (Ref.)	1 (Ref.)	1 (Ref.)	
35–49	0.76 (0.64–0.90)**	0.50 (0.39–0.65)**	1.55 (1.12–2.13)**	1.03 (0.64–1.64)	0.33 (0.27–0.40)**
25–34	0.60 (0.49–0.74)**	0.07 (0.03–0.16)**	1.39 (0.98–1.97)	0.76 (0.43–1.33)	0.14 (0.10–0.19)**
Sex					
Female	1 (Ref.)	1 (Ref.)	1 (Ref.)	1 (Ref.)	1 (Ref.)
Male	1.37 (1.16–1.61)**	1.20 (0.92–1.56)	1.54 (1.19–1.99)**	1.17 (0.77–1.80)	1.33 (1.09–1.61)**
Immigrant background					
Native	1 (Ref.)	1 (Ref.)	1 (Ref.)	1 (Ref.)	1 (Ref.)
1st generation	0.76 (0.61–0.95)*	0.74 (0.51–1.06)	0.69 (0.49–0.96)*	1.12 (0.67–1.87)	0.95 (0.74–1.20)
2nd generation	1.01 (0.83–1.22)	0.97 (0.68–1.38)	1.01 (0.78–1.31)	1.01 (0.60–1.68)	0.76 (0.57–1.00)
Onset age of regular drug use (years)					
No regular drug use	1 (Ref.)	1 (Ref.)	1 (Ref.)	1 (Ref.)	1 (Ref.)
Below 15	1.43 (1.11–1.83)*	1.22 (0.81–1.86)	4.04 (2.52–6.46)**	1.54 (0.75–3.18)	0.87 (0.63–1.19)
15–17	1.25 (0.97–1.62)	0.84 (0.51–1.37)	3.74 (2.34–5.96)**	1.34 (0.62–2.87)	0.95 (0.70–1.30)
18–24	1.27 (1.01–1.60)*	0.84 (0.53–1.35)	3.65 (2.33–5.73)**	1.61 (0.88–2.92)	0.97 (0.72–1.30)
Above 24	1.23 (1.00–1.52)	0.96 (0.70–1.31)	3.46 (2.20–5.45)**	1.66 (0.96–2.87)	1.02 (0.81–1.27)
Onset age of heavy drinking (years)					
No heavy drinking	1 (Ref.)	1 (Ref.)	1 (Ref.)	1 (Ref.)	1 (Ref.)
Below 15	0.96 (0.69–1.32)	0.85 (0.39–1.86)	1.02 (0.69–1.51)	0.65 (0.25–1.72)	0.90 (0.56–1.47)
15–17	1.03 (0.79–1.33)	0.86 (0.48–1.53)	1.05 (0.76–1.45)	1.09 (0.58–2.06)	1.05 (0.74–1.47)
18–24	0.85 (0.67–1.08)	0.79 (0.49–1.27)	0.89 (0.65–1.21)	0.94 (0.51–1.70)	1.05 (0.79–1.40)
Above 24	1.27 (1.02–1.57)*	1.43 (0.98–2.09)	0.95 (0.68–1.34)	1.29 (0.74–2.24)	1.23 (0.96–1.56)
ASI composite scores					
Mental health	1.27 (0.89–1.79)	0.49 (0.27–0.89)*	1.66 (1.00–2.75)	5.29 (2.26–12.37)**	0.54 (0.34–0.84)**
Family/social relations	0.55 (0.38–0.78)**	0.53 (0.28–0.98)*	0.63 (0.38–1.03)	0.40 (0.17–0.96)*	0.54 (0.35–0.83)**
Legal problems	0.92 (0.65–1.32)	0.22 (0.08–0.60)**	1.16 (0.74–1.79)	1.13 (0.43–3.00)	0.70 (0.41–1.18)
Drug	2.98 (1.67–5.33)**	0.06 (0.01–0.34)**	4.71 (2.23–9.96)**	2.09 (0.43–10.28)	0.99 (0.41–2.40)
Alcohol	1.37 (1.07–1.77)*	2.35 (1.57–3.53)**	1.09 (0.75–1.59)	0.79 (0.40–1.57)	0.89 (0.65–1.21)
Employment	1.79 (1.36–2.35)**	2.66 (1.79–3.95)**	2.46 (1.53–3.94)**	0.40 (0.22–0.75)**	1.78 (1.31–2.42)**
Physical health	1.34 (1.09–1.65)*	1.39 (1.01–1.91)*	1.37 (1.01–1.86)*	1.09 (0.60–1.96)	1.88 (1.46–2.42)**
Cases	12,872	12,872	12,872	12,872	12,872

*p < 0.05; **p < 0.01; ***p < 0.001.

ASI =  Addiction Severity Index.

Higher education was associated with higher risk of despair-related mortality (tertiary education: adjusted SHR = 1.45; 95% confidence interval (CI) = 1.16–1.82), alcohol-related mortality (tertiary education: adjusted SHR = 1.46; 95% CI = 1.06–2.01) and drug-related mortality (tertiary education: adjusted SHR = 1.63; 95% CI = 1.13–2.34). No other significant association was observed between education level and suicide or non-despair-related mortality.

### Sensitivity analysis

In the first set of sensitivity analyses, we replaced the categorical variable for education with the numeric measure of years of education (see supplemental Table A2). The results indicated a significant positive association between additional years of education and drug-related mortality, but no significant association for other mortality outcomes.

Next, we conducted subgroup analyses stratified by age group (see supplemental Tables A3 to A5). In line with the main findings, tertiary education was associated with a higher risk of despair-related mortality in both the youngest (25–35 years) and oldest (>50 years) age groups, as well as with alcohol-related mortality in the oldest age group (>50 years). These age-stratified results suggest that higher education may confer varying levels of risk across different life stages, potentially reflecting differences in substance use patterns, comorbidities or treatment pathways among younger and older adults. Consistent with previous research (Jemberie et al., 2023; Lundgren et al., 2022; Scarpa et al., 2023), our findings indicate that older adults are more susceptible to alcohol-related health complications, whereas younger adults are more vulnerable to drug-related harms. However, within these subgroups, individuals with tertiary education appear more prone to despair-related mortality.

## Conclusions

Previous research suggested that individuals with lower educational attainment face an increased risk of deaths of despair (Case & Deaton, 2020; Fishman & Gutin, 2021; Lübker & Murtin, 2024). Consequently, higher levels of education, which generally ensure better placement in the labour market, are considered a protective factor (Case & Deaton, 2022). These findings, primarily originating from the USA, have informed the understanding of issues such as drug use-related mortality in different national contexts, including Sweden (Ågren & Bremberg, 2023). Limited evidence from population-wide studies based on Swedish data (Ågren & Bremberg, 2022; Dahlman et al., 2021; Högberg et al., 2025) and reports from Swedish governmental agencies (Folkhälsomyndigheten et al., 2022) suggest that Swedish young adults with lower education may be similarly susceptible to drug use-related mortality, an issue that has become more concerning due to an increase in recent years.

By contrast to these population-wide studies, this study suggests that ASI assessed individuals with higher educational levels have a greater risk of mortality from alcohol- and drug-related causes compared to those with compulsory or lower education. This counterintuitive finding may be attributed to the specific nature of our sample, drawn from ASI assessments conducted by municipal social welfare offices. The selection processes determining who undergoes municipal ASI assessments in Sweden might explain why, contrary to expectations, more highly educated clients face a greater risk of despair-related mortality. These selection processes likely introduce selection bias, a form of collider bias, which leads to associations between exposures and outcomes that do not reflect broader societal trends (Munafò et al., 2018; Nilsson et al., 2020; Richiardi et al., 2019).

The literature on addiction treatment indicates that demographic characteristics, such as age, gender and ethnicity, as well as socioeconomic status, including education, influence both access to and efficacy of addiction treatments due to factors such as institutional bias, legal constraints, discrimination and individual treatment adherence (Bensley et al., 2017; Green et al., 2002; Kelly et al., 2005; Lê Cook & Alegría, 2011; Lo & Cheng, 2011; Mennis & Stahler, 2016; Scarpa et al., 2023). To our knowledge, the role of socioeconomic factors, particularly education, with respect to influencing treatment pathways for individuals with substance use disorders is under-researched in Sweden. A study specifically investigating pharmacological interventions for alcohol dependency found that individuals with lower income and educational levels were less likely to be prescribed pharmacotherapeutic treatments (Finn et al., 2021). A study by Dorner & Mittendorfer-Rutz (2017) showed that socioeconomic disparities influence both access to and outcomes of mental health treatments. The same study indicated that individuals with higher education are less likely to receive specialized healthcare and treatment with combined psychiatric medication, which are services more commonly accessed by their lower-educated counterparts, but are more likely to engage in intensive mental health care and face increased risks of suicide following such treatments.

Drawing from these insights, we hypothesize that highly educated individuals with substance use problems, equipped with greater economic resources and higher health literacy, have access to a wider range of treatment options, including healthcare services and private providers such as psychotherapists (Jemberie et al., 2023). By contrast, in Sweden, substance use treatment is mostly provided by municipal social services, which typically serve individuals facing complex psychosocial problems. Consequently, receiving support from these services is often perceived as stigmatizing due to a perceived loss of autonomy (Marttila et al., 2010). This stigma could lead highly educated individuals to consider these services only as a last resort, likely after exhausting other options or when facing more severe or complex problems (e.g., co-occurring physical and/or mental health problems). Indeed, a previous study found that only a small minority of highly educated individuals prefer municipal social services for the treatment of alcohol-related problems (Andréasson et al., 2013). Given this context, highly educated clients of municipal social services likely represent a particularly vulnerable subgroup, less able to leverage their educational advantages in managing their substance use problems.

This may explain why our results concerning the drug and alcohol use-related mortality risk of highly educated clients diverge from those drawn from population-wide studies. Hence, the collider bias in our estimates likely stems from a selection mechanism where highly educated clients access municipal social services due to severe conditions, presenting a set of characteristics that are not representative of the broader population. While our hypothesis is plausible, it remains speculative without further empirical validation. Therefore, future research should more thoroughly investigate how differences in socioeconomic background, including education, sort individuals with substance use problems into various treatment options in Sweden, as well as explore the health-related implications of these different pathways. Moreover, there are methods available to correct for sample selectivity, which could be employed to mitigate selection bias in studies focusing on treatment-seeking populations (Semykina & Wooldridge, 2018).

While we acknowledge potential collider bias as a limitation of our study, the identification of such bias in Swedish ASI assessment data highlights the need for further research, which is essential for informing the practices of professionals across various support services working with this treatment-seeking population. In Sweden, there is a strong emphasis on encouraging professionals working with clients with substance use problems to integrate evidence-based findings from academic research into their practices. However, much of this knowledge derives from population-wide studies or representative samples, which may not fully capture the complexities of the clients that professionals encounter. This discrepancy highlights the importance of conducting parallel studies on clinical samples, such as the ASI data, alongside analyses of population data from groups presenting similar issues.

Our findings also highlight the need to examine the pathways through which highly educated individuals with substance use problems access different types of services. Gaining insight into these pathways can uncover hidden vulnerabilities within seemingly advantaged groups and help tailor interventions more effectively, thereby enhancing efforts to support their recovery and well-being.

## Supplemental Material

sj-docx-1-nad-10.1177_14550725251326757 - Supplemental material for Educational attainment and deaths of despair among individuals assessed for substance use severity: Findings from Swedish Addiction Severity Index (ASI) data Supplemental material, sj-docx-1-nad-10.1177_14550725251326757 for Educational attainment and deaths of despair among individuals assessed for substance use severity: Findings from Swedish Addiction Severity Index (ASI) data by Simone Scarpa, Wossenseged Birhane Jemberie, Björn Högberg and Lena Lundgren in Nordic Studies on Alcohol and Drugs
