# Supplementary material for: Educational attainment and deaths of despair among individuals assessed for substance use severity: Findings from Swedish Addiction Severity Index (ASI) data
Source: Nordisk Alkohol Nark. 2025 Mar 13;42(5-6):479–98. doi: 10.1177/14550725251326757 (PMC11907572; doi:10.1177/14550725251326757)
Supplement: sj-docx-1-nad-10.1177_14550725251326757 - Supplemental material for Educational attainment and deaths of despair among individuals assessed for substance use severity: Findings from Swedish Addiction Severity Index (ASI) data [file sj-docx-1-nad-10.1177_14550725251326757.docx]

**Appendix**

**Table A1. Crude Fine-Gray subdistribution hazard models from the bivariate analyses (SHRs with 95% CIs).**

| **Variables** | | Total despair-related mortality | | Despair-related mortality subtypes | | | | | | Non-despair-related mortality |
| --- | --- | --- | --- | --- | --- | --- | --- | --- | --- | --- |
|  |  |  |  | Alcohol-related mortality | | Drug use-related mortality | | Suicide mortality | |  |
| *Eduction Level (n = 18,998)* | |  | |  | |  | |  | |  |
| Compulsory or below | | 1(Ref.) | | 1(Ref.) | | 1(Ref.) | | 1(Ref.) | | 1(Ref.) |
| Upper-secondary | | 1.02 (0.90-1.15) | | 1.10 (0.90-1.35) | | 0.90 (0.76-1.08) | | 1.25 (0.90-1.73) | | 0.72 (0.63-0.84)*** |
| Tertiary | | 1.33 (1.13-1.58)** | | 1.98 (1.54-2.53)*** | | 0.84 (0.64-1.11) | | 1.32 (0.76-2.28) | | 0.94 (0.77-1.15) |
| *Years of Education (n = 21,569)* | | 1.00 (0.98-1.02) | | 0.99 (0.97-1.02) | | 0.99 (0.97-1.02) | | 1.48 (0.95-2.31) | | 0.95 (0.93-0.97)*** |
| *Age group (years) n = 22,324* | |  | |  | |  | |  | |  |
| 50 yrs or older | | 1(Ref.) | | 1(Ref.) | | 1(Ref.) | | 1(Ref.) | | 1(Ref.) |
| 35-49 | | 0.71 (0.64-0.80)*** | | 0.33 (0.28-0.39)*** | | 1.91 (1.55-2.35)*** | | 1.39 (0.99-1.94) | | 0.26 (0.23-0.30)*** |
| 25-34 | | 0.62 (0.55-0.70)*** | | 0.03 (0.02-0.05)*** | | 2.60 (2.11-3.20)*** | | 1.38 (0.97-1.96) | | 0.08 (0.06-0.10)*** |
| *Sex (n = 22,324)* | |  | |  | |  | |  | |  |
| Female | | 1(Ref.) | | 1(Ref.) | | 1(Ref.) | | 1(Ref.) | | 1(Ref.) |
| Male | | 1.44 (1.28-1.62)*** | | 1.31 (1.10-1.57)** | | 1.76 (1.46-2.12)*** | | 0.98 (0.73-1.31) | | 1.46 (1.28-1.67)*** |
| *Immigrant Background (n = 21,793)* | |  | |  | |  | |  | |  |
| Native | | 1(Ref.) | | 1(Ref.) | | 1(Ref.) | | 1(Ref.) | | 1(Ref.) |
| 1^st^ generation | | 0.71 (0.61-0.83)*** | | 0.68 (0.54-0.87)** | | 0.73 (0.58-0.93)* | | 0.79 (0.52-1.19) | | 0.99 (0.85-1.15) |
| 2^nd^ generation | | 0.84 (0.73-0.98)* | | 0.50 (0.37-0.65)*** | | 1.16 (0.95-1.41) | | 1.03 (0.71-1.50) | | 0.56 (0.45-0.68)*** |
| *Onset Age of Regular Drug Use (years) n = 20,438* | |  | |  | |  | |  | |  |
| No regular drug use | | 1(Ref.) | | 1(Ref.) | | 1(Ref.) | | 1(Ref.) | | 1(Ref.) |
| below 15 | | 1.26 (1.08-1.47)** | | 0.29 (0.22-0.40)*** | | 7.36 (5.43-9.97)*** | | 1.33 (0.82-2.14) | | 0.52 (0.43-0.63)*** |
| 15-17 | | 1.10 (0.94-1.28) | | 0.21 (0.15-0.29)*** | | 6.56 (4.84-8.89)*** | | 1.37 (0.87-2.17) | | 0.46 (0.38-0.56)*** |
| 18-24 | | 1.06 (0.91-1.24) | | 0.24 (0.18-0.33)*** | | 5.63 (4.15-7.64)*** | | 1.80 (1.19-2.72)** | | 0.41 (0.34-0.50)*** |
| above 24 | | 1.21 (1.04-1.41)* | | 0.66 (0.53-0.81)*** | | 3.82 (2.75-5.30)*** | | 2.11(1.40-3.17)*** | | 0.83 (0.71-0.97)* |
| *Onset Age of Heavy Drinking (years) n = 20,398* | |  | |  | |  | |  | |  |
| No heavy drinking | | 1(Ref.) | | 1(Ref.) | | 1(Ref.) | | 1(Ref.) | | 1(Ref.) |
| below 15 | | 1.03 (0.79-1.35) | | 0.94 (0.48-1.85) | | 1.02 (0.74–1.41) | | 0.86 (0.39–1.90) | | 0.88 (0.58-1.34) |
| 15-17 | | 0.99 (0.79-1.23) | | 0.87 (0.51-1.47) | | 0.98 (0.75–1.29) | | 1.02 (0.58–1.79) | | 1.14 (0.86-1.51) |
| 18-24 | | 0.86 (0.70-1.04) | | 1.07 (0.71-1.61) | | 0.79 (0.61–1.02) | | 0.91 (0.56–1.48) | | 1.00 (0.79-1.28) |
| above 24 | | 1.28 (1.08-1.52)** | | 1.87 (1.35-2.58)*** | | 0.86 (0.66–1.12) | | 1.28 (0.81–2.03) | | 1.08 (0.88-1.32) |
| *ASI composite scores* |  | |  | |  | |  | |  | |
| Mental health (*n = 20,334*) | 1.19 (0.97–1.47) | | 0.20 (0.14–0.28)*** | | 3.01 (2.24–4.06)*** | | 7.31 (4.33–12.33)*** | | 0.24 (0.18–0.31)*** | |
| Family/social relations (*n = 20,635*) | 0.69 (0.55–0.87)** | | 0.20 (0.13–0.30)*** | | 1.48 (1.07–2.06)* | | 1.56 (0.91–2.66) | | 0.21 (0.16–0.29)*** | |
| Legal problems (n = *20,716*) | 0.97 (0.76–1.24) | | 0.05 (0.03–0.11)*** | | 3.41 (2.58–4.50)*** | | 1.22 (0.65–2.29) | | 0.28 (0.19–0.41)*** | |
| Drug (*n = 19,090*) | 1.38 (0.98–1.95) | | 0.00 (0.00–0.01)*** | | 26.45 (17.96–38.94)*** | | 3.00 (1.25–7.22)** | | 0.06 (0.04–0.11)*** | |
| Alcohol (*n = 20,006*) | 1.31 (1.11–1.54)** | | 3.79 (3.02–4.77)*** | | 0.47 (0.36–0.63)*** | | 0.97 (0.61–1.53) | | 1.62 (1.35–1.94)*** | |
| Employment (*n = 20,725*) | 1.63 (1.36–1.95)*** | | 1.00 (0.78–1.28) | | 4.28 (3.04–6.03)*** | | 0.61 (0.40–0.93)* | | 1.33 (1.10–1.62)** | |
| Physical health (*n = 21,517*) | 1.58 (1.38–1.81)*** | | 1.60 (1.30–1.97)*** | | 1.63 (1.33–2.01)*** | | 1.20 (0.81–1.76) | | 1.70 (1.46–1.99)*** | |

*: p < 0.05; **: p < 0.01; ***: p < 0.001

**Table A2. Adjusted Fine-Gray subdistribution hazard models with *Years of Education* (aSHRs with 95% CIs).**

| **Variables** | Despair-related mortality | Despair-related mortality subtypes | | | Non-despair-related mortality |
| --- | --- | --- | --- | --- | --- |
|  |  | Alcohol-related mortality | Drug use-related mortality | Suicide mortality |  |
| *Years of Education* | 1.02 (1.00-1.04) | 1.02 (0.98-1.05) | 1.04 (1.00-1.08)* | 0.99 (0.93-1.05) | 0.99 (0.96-1.01) |
| *Age group (years)* |  |  |  |  |  |
| 50 yrs or older | 1 (Ref.) | 1 (Ref.) | 1 (Ref.) | 1 (Ref.) |  |
| 35-49 | 0.74 (0.64-0.86)** | 0.53 (0.42-0.65)** | 1.43 (1.08-1.90)* | 1.12 (0.73-1.72) | 0.33 (0.28-0.39)** |
| 25-34 | 0.61 (0.51-0.73)** | 0.10 (0.05-0.18)** | 1.33 (0.98-1.81) | 0.90 (0.53-1.53) | 0.12 (0.09-0.16)** |
| *Sex* |  |  |  |  |  |
| Female | 1 (Ref.) | 1 (Ref.) | 1 (Ref.) | 1 (Ref.) | 1 (Ref.) |
| Male | 1.39 (1.20-1.61)** | 1.24 (0.99-1.55) | 1.65 (1.31-2.09)** | 1.01 (0.70-1.47) | 1.43 (1.20-1.70)** |
| *Immigrant Background* |  |  |  |  |  |
| Native | 1 (Ref.) | 1 (Ref.) | 1 (Ref.) | 1 (Ref.) | 1 (Ref.) |
| 1^st^ generation | 0.77 (0.64-0.93)** | 0.81 (0.61-1.07) | 0.68 (0.50-0.91)** | 1.07 (0.67-1.71) | 0.94 (0.77-1.14) |
| 2^nd^ generation | 0.89 (0.75-1.07) | 0.74 (0.53-1.04) | 0.93 (0.73-1.18) | 1.05 (0.66-1.68) | 0.74 (0.58-0.95)* |
| *Onset Age of Regular Drug Use (years)* |  |  |  |  |  |
| No regular drug use | 1 (Ref.) | 1 (Ref.) | 1 (Ref.) | 1 (Ref.) | 1 (Ref.) |
| below 15 | 1.28 (1.03-1.59)* | 1.01 (0.70-1.47) | 3.50 (2.34-5.24)** | 1.43 (0.74-2.76) | 0.92 (0.71-1.19) |
| 15-17 | 1.16 (0.93-1.44) | 0.87 (0.59-1.29) | 3.19 (2.13-4.77)** | 1.30 (0.67-2.53) | 0.89 (0.69-1.16) |
| 18-24 | 1.19 (0.97-1.46) | 0.78 (0.53-1.16) | 3.30 (2.23-4.88)** | 1.52 (0.89-2.60) | 0.89 (0.69-1.15) |
| above 24 | 1.20 (0.99-1.44) | 1.06 (0.82-1.37) | 2.87 (1.93-4.27)** | 1.65 (1.00-2.73) | 0.99 (0.81-1.21) |
| *Onset Age of Heavy Drinking (years)* |  |  |  |  |  |
| No heavy drinking | 1 (Ref.) | 1 (Ref.) | 1 (Ref.) | 1 (Ref.) | 1 (Ref.) |
| below 15 | 0.94 (0.72-1.22) | 0.91 (0.51-1.63) | 1.04 (0.75-1.44) | 0.64 (0.30-1.38) | 1.04 (0.74-1.48) |
| 15-17 | 1.07 (0.86-1.34) | 1.00 (0.64-1.57) | 1.15 (0.87-1.53) | 0.92 (0.53-1.59) | 1.10 (0.83-1.45) |
| 18-24 | 0.88 (0.71-1.08) | 0.92 (0.62-1.38) | 0.93 (0.70-1.24) | 0.71 (0.41-1.23) | 1.06 (0.82-1.36) |
| above 24 | 1.26 (1.04-1.53)* | 1.41 (1.01-1.97)* | 1.03 (0.75-1.40) | 1.04 (0.63-1.72) | 1.25 (1.00-1.55)* |
| *ASI composite scores* |  |  |  |  |  |
| Mental health | 1.27 (0.95-1.71) | 0.57 (0.35-0.94)* | 1.74 (1.13-2.68)* | 4.26 (1.91-9.50)** | 0.63 (0.44-0.92)* |
| Family/social relations | 0.60 (0.44-0.82)** | 0.50 (0.30-0.85)** | 0.67 (0.43-1.06) | 0.63 (0.28-1.40) | 0.48 (0.33-0.72)** |
| Legal problems | 0.95 (0.70-1.30) | 0.34 (0.16-0.75)** | 1.13 (0.76-1.67) | 1.20 (0.50-2.92) | 0.81 (0.52-1.26) |
| Drug | 1.95 (1.15-3.28)* | 0.02 (0.00-0.11)** | 3.95 (2.03-7.66)** | 1.37 (0.31-5.98) | 1.05 (0.49-2.27) |
| Alcohol | 1.29 (1.03-1.60)* | 2.30 (1.64-3.22)** | 0.90 (0.64-1.27) | 0.80 (0.43-1.48) | 1.00 (0.78-1.29) |
| Employment | 1.77 (1.39-2.25)** | 2.45 (1.74-3.45)** | 2.35 (1.54-3.59)** | 0.40 (0.23-0.70)** | 1.82 (1.39-2.39)** |
| Physical health | 1.34 (1.12-1.61)** | 1.40 (1.07-1.84)* | 1.28 (0.98-1.68) | 1.24 (0.73-2.09) | 1.64 (1.33-2.02)** |
| Cases | 14,668 | 14,668 | 14,668 | 14,668 | 14,668 |

*: p < 0.05; **: p < 0.01; ***: p < 0.001

**Table A3. Adjusted Fine-Gray subdistribution hazard models, individuals aged 25-34 (aSHRs with 95% CIs).**

| **Variables** | Despair-related mortality | Despair-related mortality subtypes^a^ | | Non-despair-related mortality |
| --- | --- | --- | --- | --- |
|  |  | Drug use-related mortality | Suicide mortality |  |
| *Education Level* |  |  |  |  |
| Compulsory or below | 1 (Ref.) | 1 (Ref.) | 1 (Ref.) | 1 (Ref.) |
| Upper-secondary | 1.23 (0.92-1.64) | 1.12 (0.81 - 1.55) | 1.54 (0.77 - 3.09) | 0.93 (0.52 - 1.67) |
| Tertiary | 1.95 (1.10-3.44)* | 1.80 (0.95 - 3.43) | 2.78 (0.79 - 9.73) | 1.13 (0.27 - 4.78) |
| *Sex* |  |  |  |  |
| Female | 1 (Ref.) | 1 (Ref.) | 1 (Ref.) | 1 (Ref.) |
| Male | 1.97 (1.35-2.89)** | 2.56 (1.62 - 4.06)*** | 0.78 (0.35 - 1.74) | 2.88 (1.05 - 7.90)* |
| *Immigrant Background* |  |  |  |  |
| Native | 1 (Ref.) | 1 (Ref.) | 1 (Ref.) | 1 (Ref.) |
| 1^st^ generation | 0.51 (0.31-0.83)** | 0.40 (0.22 - 0.74)** | 1.07 (0.41 - 2.76) | 1.45 (0.67 - 3.15) |
| 2^nd^ generation | 0.90 (0.64-1.27) | 0.87 (0.60 - 1.26) | 0.95 (0.40 - 2.27) | 1.26 (0.60 - 2.62) |
| *Onset Age of Regular Drug Use (years)* |  |  |  |  |
| No regular drug use | 1 (Ref.) | 1 (Ref.) | 1 (Ref.) | 1 (Ref.) |
| below 15 | 3.47 (1.49-8.07)** | 5.57 (1.61 - 19.27)** | 2.95 (0.52 - 16.75) | 2.09 (0.64 - 6.78) |
| 15-17 | 2.76 (1.20-6.31)* | 4.97 (1.46 - 16.92)* | 1.28 (0.24 - 6.88) | 1.36 (0.42 - 4.39) |
| 18-24 | 3.24 (1.45-7.26)** | 5.26 (1.58 - 17.58)** | 3.02 (0.64 - 14.37) | 1.08 (0.33 - 3.54) |
| above 24 | 2.45 (0.97-6.19) | 4.49 (1.23 - 16.44)* | 1.01 (0.13 - 8.06) | 0.97 (0.17 - 5.45) |
| *Onset Age of Heavy Drinking (years)* |  |  |  |  |
| No heavy drinking | 1 (Ref.) | 1 (Ref.) | 1 (Ref.) | 1 (Ref.) |
| below 15 | 0.91 (0.56-1.48) | 1.08 (0.64 - 1.81) | 0.41 (0.08 - 2.10) | 0.85 (0.30 - 2.44) |
| 15-17 | 1.10 (0.75-1.61) | 1.10 (0.72 - 1.70) | 1.17 (0.49 - 2.82) | 0.85 (0.35 - 2.06) |
| 18-24 | 0.89 (0.61-1.30) | 0.97 (0.64 - 1.48) | 0.66 (0.24 - 1.79) | 0.76 (0.34 - 1.68) |
| above 24 | 1.01 (0.56-1.83) | 0.84 (0.39 - 1.78) | 1.65 (0.57 - 4.79) | 0.68 (0.19 - 2.47) |
| *ASI composite scores* |  |  |  |  |
| Mental health | 3.07 (1.61-5.85)** | 2.17 (1.02 - 4.58)* | 12.33 (3.39 - 44.83)*** | 0.24 (0.05 - 1.07) |
| Family/social relations | 0.48 (0.24-0.97)* | 0.56 (0.26 - 1.24) | 0.21 (0.04 - 1.01) | 0.28 (0.07 - 1.12) |
| Legal problems | 0.99 (0.55-1.79) | 1.09 (0.58 - 2.05) | 0.76 (0.14 - 4.00) | 5.68 (2.43 - 13.28)*** |
| Drug | 4.37 (1.66-11.51)** | 4.46 (1.55 - 12.82)** | 6.44 (0.46 - 89.32) | 0.49 (0.08 - 3.16) |
| Alcohol | 0.97 (0.55-1.71) | 0.85 (0.44 - 1.63) | 1.09 (0.32 - 3.69) | 3.64 (1.12 - 11.88)* |
| Employment | 1.34 (0.74-2.43) | 1.69 (0.83 - 3.42) | 0.51 (0.15 - 1.82) | 4.73 (0.94 - 23.78) |
| Physical health | 1.26 (0.82-1.94) | 1.30 (0.82 - 2.08) | 1.11 (0.34 - 3.62) | 1.67 (0.67 - 4.19) |
| Cases | 4,629 | 4,629 | 4,629 | 4,629 |

^a^: Because no cases of alcohol-related mortality occurred among individuals aged 25–34 with tertiary education, the model for this outcome was not estimated for this subgroup.

*: p < 0.05; **: p < 0.01; ***: p < 0.001

**Table A4. Adjusted Fine-Gray subdistribution hazard models, individuals aged 35-49 (aSHRs with 95% CIs).**

| **Variables** | Despair-related mortality | Despair-related mortality subtypes | | | Non-despair-related mortality |
| --- | --- | --- | --- | --- | --- |
|  |  | Alcohol-related mortality | Drug use-related mortality | Suicide mortality |  |
| *Education Level* |  |  |  |  |  |
| Compulsory or below | 1 (Ref.) | 1 (Ref.) | 1 (Ref.) | 1 (Ref.) | 1 (Ref.) |
| Upper-secondary | 1.14 (0.88-1.47) | 1.03 (0.64 - 1.64) | 1.16 (0.82 - 1.65) | 1.16 (0.63 - 2.12) | 1.08 (0.75 - 1.56) |
| Tertiary | 1.23 (0.83-1.83) | 0.88 (0.43 - 1.80) | 1.59 (0.91 - 2.77) | 1.13 (0.44 - 2.87) | 0.93 (0.51 - 1.70) |
| *Sex* |  |  |  |  |  |
| Female | 1 (Ref.) | 1 (Ref.) | 1 (Ref.) | 1 (Ref.) | 1 (Ref.) |
| Male | 1.32 (1.00-1.73)* | 1.18 (0.73 - 1.89) | 1.39 (0.93 - 2.07) | 1.32 (0.69 - 2.52) | 1.53 (1.03 - 2.29)* |
| *Immigrant Background* |  |  |  |  |  |
| Native | 1 (Ref.) | 1 (Ref.) | 1 (Ref.) | 1 (Ref.) | 1 (Ref.) |
| 1^st^ generation | 0.80 (0.55-1.14) | 0.56 (0.27 - 1.16) | 0.79 (0.48 - 1.32) | 1.31 (0.62 - 2.77) | 0.68 (0.40 - 1.14) |
| 2^nd^ generation | 1.03 (0.76-1.40) | 1.08 (0.63 - 1.86) | 0.98 (0.64 - 1.50) | 1.04 (0.48 - 2.27) | 0.65 (0.39 - 1.08) |
| *Onset Age of Regular Drug Use (years)* |  |  |  |  |  |
| No regular drug use | 1 (Ref.) | 1 (Ref.) | 1 (Ref.) | 1 (Ref.) | 1 (Ref.) |
| below 15 | 1.85 (1.22-2.80)** | 1.17 (0.54 - 2.51) | 3.89 (1.95 - 7.75)*** | 1.79 (0.62 - 5.16) | 0.85 (0.44 - 1.66) |
| 15-17 | 1.63 (1.07-2.49)* | 1.13 (0.55 - 2.34) | 3.26 (1.65 - 6.42)*** | 2.10 (0.68 - 6.50) | 1.15 (0.64 - 2.09) |
| 18-24 | 1.17 (0.79-1.74) | 0.56 (0.25 - 1.28) | 2.81 (1.46 - 5.43)** | 1.19 (0.45 - 3.14) | 1.09 (0.63 - 1.88) |
| above 24 | 1.37 (0.96-1.94) | 0.88 (0.49 - 1.57) | 2.75 (1.46 - 5.17)** | 1.79 (0.81 - 3.98) | 1.13 (0.70 - 1.82) |
| *Onset Age of Heavy Drinking (years)* |  |  |  |  |  |
| No heavy drinking | 1 (Ref.) | 1 (Ref.) | 1 (Ref.) | 1 (Ref.) | 1 (Ref.) |
| below 15 | 0.90 (0.52-1.57) | 0.47 (0.06 - 3.74) | 0.96 (0.50 - 1.86) | 0.90 (0.25 - 3.27) | 0.79 (0.29 - 2.17) |
| 15-17 | 0.86 (0.55-1.36) | 1.48 (0.49 - 4.53) | 0.78 (0.44 - 1.39) | 0.77 (0.26 - 2.29) | 1.23 (0.64 - 2.38) |
| 18-24 | 0.86 (0.57-1.31) | 1.57 (0.61 - 4.00) | 0.78 (0.45 - 1.34) | 0.80 (0.29 - 2.23) | 1.24 (0.69 - 2.22) |
| above 24 | 1.31 (0.92-1.88) | 3.10 (1.35 - 7.11)* | 1.02 (0.62 - 1.69) | 0.86 (0.36 - 2.07) | 1.35 (0.80 - 2.25) |
| *ASI composite scores* |  |  |  |  |  |
| Mental health | 0.99 (0.54-1.82) | 0.36 (0.12 - 1.07) | 1.26 (0.53 - 2.96) | 2.96 (0.73 - 12.02) | 0.55 (0.23 - 1.31) |
| Family/social relations | 0.63 (0.36-1.10) | 0.51 (0.16 - 1.59) | 0.84 (0.40 - 1.77) | 0.39 (0.10 - 1.49) | 1.77 (0.81 - 3.88) |
| Legal problems | 1.16 (0.66-2.03) | 0.17 (0.03 - 0.96)* | 1.88 (0.97 - 3.65) | 0.85 (0.19 - 3.86) | 0.56 (0.20 - 1.53) |
| Drug | 2.26 (0.85-6.00) | 0.25 (0.02 - 2.54) | 3.51 (1.00 - 12.31)* | 1.30 (0.09 - 18.12) | 0.74 (0.16 - 3.50) |
| Alcohol | 1.47 (0.97-2.24) | 2.68 (1.25 - 5.78)* | 1.19 (0.67 - 2.12) | 1.05 (0.34 - 3.26) | 0.81 (0.43 - 1.52) |
| Employment | 1.82 (1.17-2.84)** | 2.18 (1.08 - 4.39)* | 3.47 (1.66 - 7.26)*** | 0.45 (0.18 - 1.13) | 1.37 (0.75 - 2.52) |
| Physical health | 1.54 (1.08-2.21)* | 1.61 (0.86 - 3.02) | 1.50 (0.91 - 2.46) | 1.42 (0.57 - 3.50) | 2.31 (1.35 - 3.94)** |
| Cases | 4,512 | 4,512 | 4,512 | 4,512 | 4,512 |

*: p < 0.05; **: p < 0.01; ***: p < 0.001

**Table A5. Adjusted Fine-Gray subdistribution hazard models, individuals aged >50 (aSHRs with 95% CIs).**

| **Variables** | Despair-related mortality | Despair-related mortality subtypes | | | Non-despair-related mortality |
| --- | --- | --- | --- | --- | --- |
|  |  | Alcohol-related mortality | Drug use-related mortality | Suicide mortality |  |
| *Education Level* |  |  |  |  |  |
| Compulsory or below | 1 (Ref.) | 1 (Ref.) | 1 (Ref.) | 1 (Ref.) | 1 (Ref.) |
| Upper-secondary | 1.15 (0.89-1.50) | 1.11 (0.81 - 1.52) | 1.38 (0.77 - 2.46) | 0.93 (0.43 - 2.01) | 0.82 (0.66 - 1.02) |
| Tertiary | 1.50 (1.10-2.05)* | 1.66 (1.16 - 2.39)** | 1.63 (0.78 - 3.40) | 0.60 (0.20 - 1.80) | 0.79 (0.59 - 1.05) |
| *Sex* |  |  |  |  |  |
| Female | 1 (Ref.) | 1 (Ref.) | 1 (Ref.) | 1 (Ref.) | 1 (Ref.) |
| Male | 1.09 (0.84-1.40) | 1.15 (0.84 - 1.58) | 0.68 (0.39 - 1.18) | 1.58 (0.72 - 3.47) | 1.22 (0.97 - 1.54) |
| *Immigrant Background* |  |  |  |  |  |
| Native | 1 (Ref.) | 1 (Ref.) | 1 (Ref.) | 1 (Ref.) | 1 (Ref.) |
| 1^st^ generation | 0.95 (0.67-1.34) | 0.82 (0.53 - 1.26) | 1.47 (0.72 - 3.00) | 0.98 (0.34 - 2.80) | 0.98 (0.73 - 1.32) |
| 2^nd^ generation | 1.06 (0.73-1.54) | 0.84 (0.51 - 1.40) | 1.94 (0.97 - 3.88) | 0.99 (0.28 - 3.47) | 0.70 (0.48 - 1.03) |
| *Onset Age of Regular Drug Use (years)* |  |  |  |  |  |
| No regular drug use | 1 (Ref.) | 1 (Ref.) | 1 (Ref.) | 1 (Ref.) | 1 (Ref.) |
| below 15 | 1.12 (0.71-1.75) | 1.20 (0.72 - 2.01) | 2.64 (0.96 - 7.22) | 0.55 (0.05 - 6.58) | 0.73 (0.47 - 1.12) |
| 15-17 | 1.08 (0.67-1.73) | 0.69 (0.34 - 1.38) | 4.22 (1.66 - 10.75)** | 1.71 (0.39 - 7.53) | 0.93 (0.61 - 1.42) |
| 18-24 | 1.26 (0.81-1.96) | 1.10 (0.63 - 1.94) | 3.92 (1.51 - 10.18)** | 1.03 (0.24 - 4.37) | 1.12 (0.76 - 1.63) |
| above 24 | 1.24 (0.92-1.66) | 0.97 (0.67 - 1.41) | 3.65 (1.65 - 8.07)*** | 2.05 (0.93 - 4.53) | 0.99 (0.76 - 1.29) |
| *Onset Age of Heavy Drinking (years)* |  |  |  |  |  |
| No heavy drinking | 1 (Ref.) | 1 (Ref.) | 1 (Ref.) | 1 (Ref.) | 1 (Ref.) |
| below 15 | 1.06 (0.52-2.16) | 1.19 (0.51 - 2.80) | 0.87 (0.22 - 3.49) | 0.00(0.00- 0.00)*** | 0.89 (0.45 - 1.75) |
| 15-17 | 1.12 (0.66-1.90) | 0.74 (0.36 - 1.52) | 1.86 (0.70 - 4.90) | 1.62 (0.27 - 9.91) | 0.90 (0.56 - 1.45) |
| 18-24 | 0.83 (0.52-1.32) | 0.63 (0.35 - 1.14) | 0.97 (0.39 - 2.41) | 2.53 (0.65 - 9.79) | 0.98 (0.67 - 1.44) |
| above 24 | 1.17 (0.81-1.67) | 1.13 (0.73 - 1.74) | 0.73 (0.34 - 1.59) | 2.52 (0.74 - 8.56) | 1.16 (0.87 - 1.56) |
| *ASI composite scores* |  |  |  |  |  |
| Mental health | 0.84 (0.47-1.52) | 0.51 (0.25 - 1.06) | 1.50 (0.46 - 4.94) | 6.17 (0.90 - 42.21) | 0.62 (0.35 - 1.11) |
| Family/social relations | 0.52 (0.28-0.96)* | 0.53 (0.25 - 1.14) | 0.35 (0.09 - 1.29) | 0.74 (0.14 - 4.02) | 0.31 (0.17 - 0.56)** |
| Legal problems | 0.32 (0.12-0.85)* | 0.28 (0.08 - 0.97)* | 0.05 (0.00 - 0.77)* | 4.75 (0.85 - 26.54) | 0.24 (0.09 - 0.62)** |
| Drug | 0.81 (0.20-3.33) | 0.03 (0.00 - 0.37)** | 16.52 (2.44 - 111.68)** | 0.52 (0.01 - 22.76) | 0.98 (0.25 - 3.80) |
| Alcohol | 1.69 (1.14-2.49)** | 2.04 (1.28 - 3.27)** | 1.79 (0.80 - 4.01) | 0.47 (0.13 - 1.74) | 0.79 (0.55 - 1.15) |
| Employment | 2.17 (1.41-3.33)** | 2.96 (1.81 - 4.85)** | 3.28 (0.97 - 11.14) | 0.25 (0.07 - 0.82)* | 1.92 (1.32 - 2.78)** |
| Physical health | 1.21 (0.87-1.68) | 1.33 (0.91 - 1.95) | 1.07 (0.49 - 2.33) | 0.81 (0.27 - 2.41) | 1.80 (1.33 - 2.44)*** |
| Cases | 3,731 | 3,731 | 3,731 | 3,731 | 3,731 |

*: p < 0.05; **: p < 0.01; ***: p < 0.001
